# Gene Expression Profiles Characterize Inflammation Stages in the Acute Lung Injury in Mice

**DOI:** 10.1371/journal.pone.0011485

**Published:** 2010-07-08

**Authors:** Isabelle Lesur, Julien Textoris, Béatrice Loriod, Cécile Courbon, Stéphane Garcia, Marc Leone, Catherine Nguyen

**Affiliations:** 1 Inserm U928, TAGC, Parc scientifique de Luminy, Université de la méditerranée, Marseille, France; 2 Service d'anesthésie et de réanimation, hôpital Nord, AP-HM, Université de la méditerranée, Marseille, France; 3 Service d'anesthésie et de réanimation, hôpital LaTimone, AP-HM, Université de la méditerranée, Marseille, France; 4 Faculté de Médecine – Secteur Nord, labo transfert d'oncologie, Université de la méditerranée, Marseille, France; University of Pittsburgh, United States of America

## Abstract

Acute Lung Injury (ALI) carries about 50 percent mortality and is frequently associated with an infection (sepsis). Life-support treatment with mechanical ventilation rescues many patients, although superimposed infection or multiple organ failure can result in death. The outcome of a patient developing sepsis depends on two factors: the infection and the pre-existing inflammation. In this study, we described each stage of the inflammation process using a transcriptional approach and an animal model. Female C57BL6/J mice received an intravenous oleic acid injection to induce an acute lung injury (ALI). Lung expression patterns were analyzed using a 9900 cDNA mouse microarray (MUSV29K). Our gene-expression analysis revealed marked changes in the immune and inflammatory response metabolic pathways, notably lipid metabolism and transcription. The early stage (1 hour–1.5 hours) is characterized by a pro-inflammatory immune response. Later (3 hours–4 hours), the immune cells migrate into inflamed tissues through interaction with vascular endothelial cells. Finally, at late stages of lung inflammation (18 hours–24 hours), metabolism is deeply disturbed. Highly expressed pro-inflammatory cytokines activate transcription of many genes and lipid metabolism. In this study, we described a global overview of critical events occurring during lung inflammation which is essential to understand infectious pathologies such as sepsis where inflammation and infection are intertwined. Based on these data, it becomes possible to isolate the impact of a pathogen at the transcriptional level from the global gene expression modifications resulting from the infection associated with the inflammation.

## Introduction

Acute lung injury (ALI) is a diffuse lung injury which is characterized by a widespread capillary leakage leading to hypoxemia and low lung compliance. ALI is caused by either a direct (e.g. pneumonia) or indirect (e.g. pancreatitis) injury of the lung. Any local (e.g. pneumonia) or systemic inflammation (e.g. pancreatitis) can lead to a critical alteration of the lung function. The Acute Respiratory Distress Syndrome (ARDS) is the last stage of this acute inflammatory process and still carries a high mortality rate (40–50%) [1]. Sepsis is a major cause of ARDS either by direct alteration of the lung or indirectly through the Systemic Inflammatory Response Syndrome (SIRS) associated with severe sepsis. Inflammation is part of the defense mechanisms of innate immunity, occurring after tissue injury. At the site of inflammation, a cascade of mediators such as cytokines initiates activation of inflammatory cells (early-inflammatory phase). Then, white blood cells migrate through the wall of blood vessels and infiltrate the surrounding tissues. ARDS frequently occurs in a context of severe sepsis in which inflammation and infection interplay. Then, it is unclear to delineate the pathways related to inflammation or infection. In sepsis, the production of both pro- and anti-inflammatory cytokines in sepsis has been widely studied [2]. While pro-inflammatory cytokines are necessary for initiating an effective inflammatory process against infection, anti-inflammatory cytokines seem to be a prerequisite for controlling and down-regulating the inflammatory response leading to a depression of the immune system of patients [3]. Human immune responses to sepsis are mediated mainly by the primary pro-inflammatory cytokines. The timing of cytokine release and the balance between pro- and anti-inflammatory mediators seems to be associated with the severity of sepsis [4]–[5]. An excessive production may induce deleterious effects [5]. To this purpose, deciphering gene expression profiles of either infection or inflammation alone seems crucial.

One way to do so consists on studying transcriptional genes expression profiles at several inflammation stages (with or without sepsis). Genome-wide gene expression profiling using microarray technology has been applied successfully to the study of human disease pathogenesis. Examples include the discovery of new cancer subtypes with different prognosis and response to therapies [6], or new hypotheses of disease pathogenesis in ARDS [7]. Gene expression analysis by microarray studies of genes clusters that have similar expression changes over time allows the definition of functionally meaningful expression patterns. This approach has been successfully used to study either ARDS, using either a whole organ or cultured cells or in severe sepsis studies where inflammation was associated with infection [8].

In humans with ARDS, time-course studies using blood samples made it possible to obtain successful results using a transcriptional approach. Wang *et al.* studied global gene expression profiling in 8 human blood samples and identified potential candidate genes that can be used as biomarkers for ARDS [9]. They reported for instance the role of peptidase inhibitor 3 (Serpina1c/PI3) encoding Elafin which has antimicrobial and anti-inflammatory activities. One limitation in using human samples is the heterogeneity of the samples with patients from different gender, age, injury, ethnic origin and genetic backgrounds.

Previous transcriptional studies performed to study ARDS used various experimental techniques, various species and various types of infection. The common point between these studies is the sequence of events: first, an infection is associated with an inflammation and then, samples are studied using a transcriptional approach. At this point, since gene expression patterns depend on both inflammation and infection, it is not possible to identify the impact of either inflammation or infection on gene expression. A similar infective agent leads to different outcomes depending on the pre-existent inflammation stage of each patient [4]. For this reason, we decided to set up a mouse model to study ALI without any infection. Inflammation induced by intravenous administration of Oleic Acid (OA) resembles ARDS in many morphological, histological, and physiological respects [10]. OA-induced ALI is consistently associated with acute respiratory failure characterized by hypoxemia and reduced lung compliance due to alveolar damage, intra-alveolar hemorrhage, and leakage of proteinaceous fluid into the air space [11]. The progression of OA-induced injury is much shorter than that of human ARDS. The acute phase of human ARDS develops over 1–7 days. However, it is also characterized by extensive damage to the alveolar epithelium, hemorrhage, and pulmonary edema. Despite some limitations, the OA-ALI remains a relevant model for investigating the effects of ALI. Indeed, no other model has been used as extensively to evaluate the risks and benefits of various treatment strategies before applying them to patients with ARDS [10].

Our objective was to identify some biologically relevant process important in ALI by studying the molecular interactions between OA and inflammation in a mouse model. The modest success of microarray approaches to identify novel potential therapeutic targets may be due to a lack of comprehension to how changes in gene expression occur through time. For this reason, we decided to investigate lung gene expression patterns in female mice developing inflammation without any infection using a 9900 cDNA mouse microarray over a 24 hours time-course. By looking at changes in gene expression over time, we developed a global strategy to identify novel pathways that could represent novel targets for therapy.

## Results

### Outcome of mice and definition of pro- and anti-inflammatory phases by real-time PCR

**Figure 1:**
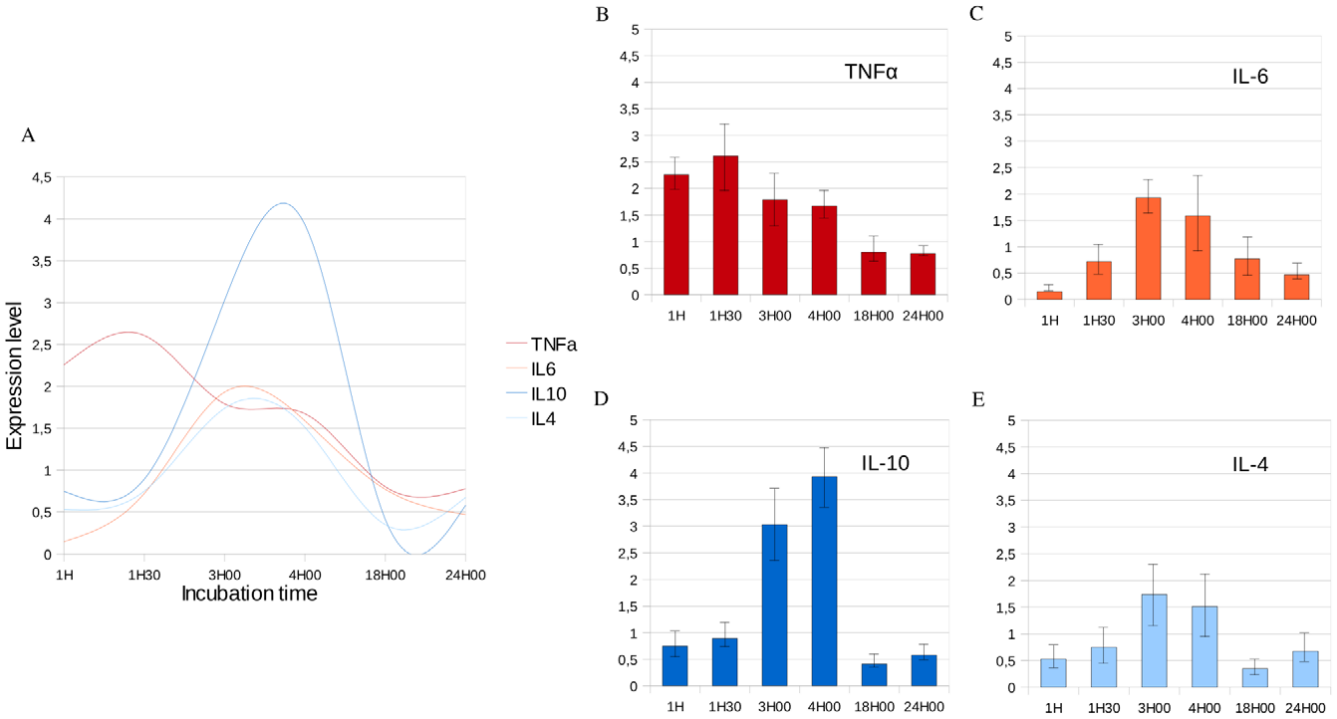
Variation of expression of pro-inflammatory and anti-inflammatory cytokines determined by real-time RT-PCR in lung samples. The X-axis corresponds to the incubation time after OA injection. The Y-axis corresponds to the ratio between the expression level of a cytokine measured after OA injection and the expression level of this same cytokine measured after the same incubation time following physiological serum injection. Measurements are normalized using the βactin housekeeping gene. A- Variations of expression level of Tnfα, Il6, Il10 and Il4 across OA incubation time allowed us to identify pro- and anti-inflammatory phases. B- We identified the pro-inflammatory peak 1H30 after injection of OA which corresponds to the maximum expression level of Tnfα. C- Il6 which is both a pro- and anti-inflammatory cytokine is highly expressed 3H after OA injection. D- The expression level of Il10, an anti-inflammatory cytokine is maximal 4H after injection of OA. E- Il4 is highly expressed 3H to 4H after injection of OA. We observe that the anti-inflammatory response occurs approximately 4H after OA injection and follows the pro-inflammatory response which occurs approximately 1H30 after OA injection.

Following the injection of OA, we noticed a transitional prostration of the mice which started 1.5 hours after injection and lasted for several hours. All mice recovered; the survival rate was 100% after 24 hours incubation. To evaluate for the incubation period necessary to induce lung inflammation, we considered a wide time-course ranging from 1 hour to 24 hours after OA injection even if previously published studies considered much shorter incubation times [12]–[13]. Prior to large-scale gene expression analysis, we quantified expression level of one pro-inflammatory cytokine (Tnfα), one cytokine with pro- and anti-inflammatory activities (Il6), and two anti-inflammatory cytokines (Il10 and Il4) at 1 hour, 1.5 hours, 3 hours, 4 hours, 18 hours and 24 hours after OA and physiological serum injection (figure 1 A). Expression levels of Tnfα, Il6, Il10 and Il4 peaked respectively at 1.5 hours, 3 hours, 4 hours and 3 hours-4 hours after OA injection. Thus, inflammatory response ranged from 60 minutes to several hours following OA injection. Real-time PCR allowed us to identify a pro-inflammatory expression peak 1.5 hours after OA injection (figure 1 B), a transition phase 3 hours to 4 hours after OA injection (figure 1 C) and an anti-inflammatory phase 4 hours after OA injection (figure 1 D, E).

### Identification of the inflammation timing following OA injection using lung histology

**Figure 2:**
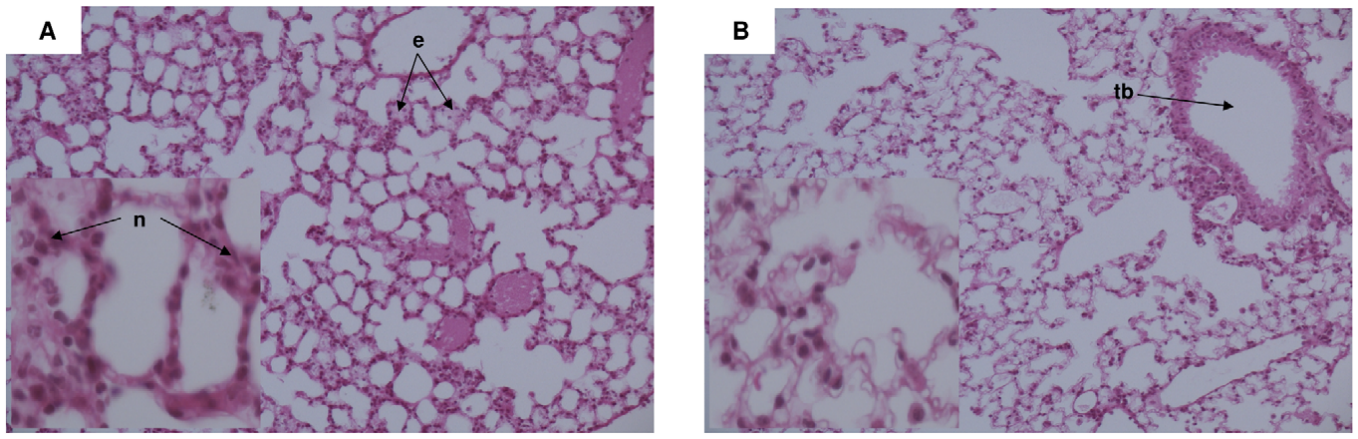
Histological quantification of lung injury following OA injection. Sections are representative of all animals examined per group. A- Section of lung 1H30 after OA injection showing interstitial neutrophils (n) and edema (e). There was no significant fibrin formation within the alveolar spaces – B- Section of lung 3H00 after OA injection showing absence of edema or fibrin. There was no more significant neutrophil infiltrate - (tb) Terminal bronchiole. (H&E x100, cartridges X400).

Compared with physiological serum controls, OA induced a transient increase in histological lung damage 1.5 hours after injection (figure 2). Histologically, 1.5 hours after OA injection, we found a neutrophil accumulation in the alveolar wall associated with edema. Lung histological modifications are thus correlated with the transcriptional up-regulation of pro-inflammatory mediators (both by microarray and RT-PCR). These results strengthened our real-time PCR data. There were no more histological lung lesions 3 hours after OA injection.

### Microarray analysis discriminated between early- and late-inflammatory stages according to the time-course after injection

**Figure 3:**
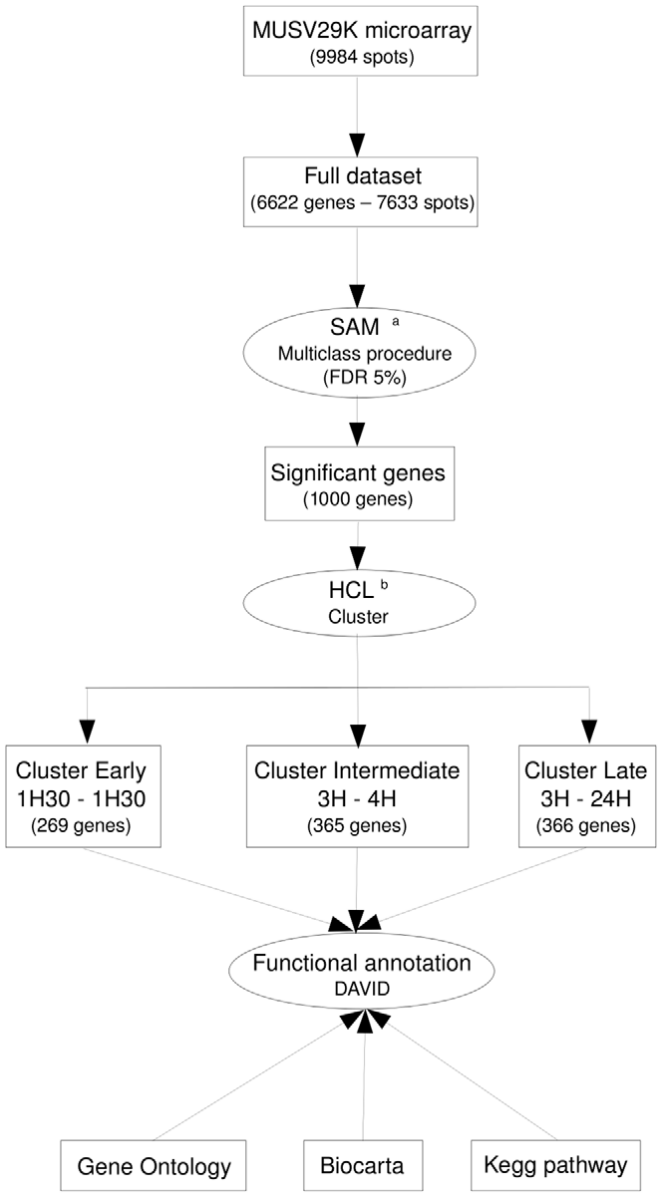
Schematic outline of data analysis. ^a^ We compared gene expression between three groups: 1H-1H30/3H-4H/18H-24H. ^b^ HCL: Hierarchical Clustering. We considered the full dataset (n = 6622 genes) to identify genes significantly differentially expressed between the three classes previously described. The 1000 genes identified with SAM have been clustered using the Cluster software and each cluster was independently functionally annotated using DAVID.

To focus on transcriptional changes associated with the inflammatory process in lung, we searched for a set of genes that discriminated between mice at different incubation times. To this purpose, we used the multi-class SAM procedure [14], and we applied a false discovery rate of 5% (figure 3 and see supplementary data file S1). The analysis yielded a set of 1000 genes (see supplementary data file S2). By performing a hierarchical clustering of the samples (microarrays), we identified 3 clusters (see supplementary data file S3). Samples at 1 hour and 1.5 hours clustered together. The same way, samples at 3 hours and 4 hours were grouped together. Finally, samples at 18 hours and 24 hours clustered together with the exception of three 18 hours samples which have been grouped with the 3 hours–4 hours cluster as an out-group.

**Figure 4:**
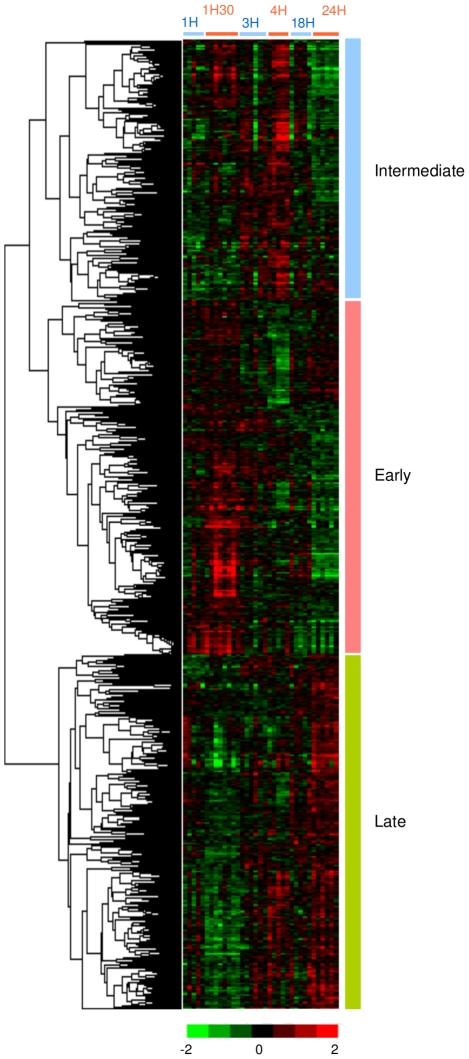
Hierarchical classification of 1000 significant genes differentially expressed at early and late stages of inflammation. This set of genes was extracted from the full dataset (n = 9984) by use of a SAM procedure and a false discovery rate of 5%. Each row represents a gene and each column represents a sample. Red and green indicate expression levels respectively above and below the median. Dendrogram of genes, to the left of the matrix represents overall similarities in gene expression profiles. 3 clusters have been identified: Early, Intermediate and Late.

We also performed unsupervised hierarchical clustering on the 1000 genes (figure 4). Interestingly, the expression level of numerous genes was induced specifically either early or late after OA injection while other genes were specifically up-regulated during the transition phase between the early- and late-inflammatory stages (see supplementary data file S4). The Cluster software [15] classified the 1000 significantly differentially expressed genes into 3 main clusters. Genes of cluster Early (365 genes) were over-expressed at early stage of inflammation (1 hour–1.5 hours); their expression level decreased at 3–4 hours and then these genes were under-expressed at late time-points (18 hours–24 hours). Genes of the Intermediate cluster (269 genes) were over-expressed between 1.5 hours and 4 hours compared to the earliest time-point (1 hour) and the late measurements (18 hours –24 hours), which correspond to the transition between early- and late-inflammatory responses. At the latest stage of inflammation, genes from the Late cluster (366 genes) were highly over-expressed. Expression level of these genes increased at 3 hours to 4 hours after OA injection and reached a peak at 18 hours–24 hours.

To analyze functional annotations related to the pulmonary inflammation, we considered biological process Gene Ontology (GO) terms, Kegg pathways and the Biocarta database using DAVID for the 365 genes belonging to the cluster Early, the 269 genes belonging to the cluster Intermediate, and the 366 genes belonging to the cluster Late.

GO terms related to the «Defense response» (p = 9.9×10^−3^) such as «Inflammatory response» (p = 6.9×10^−3^), “cytokine activity” (p = 1.1×10^−2^) and «Immune response» (p = 6.5×10^−3^) were strongly represented in cluster Early (table 1). Similarly, several genes grouped in the cluster Early were found to be involved in Kegg pathways related to immune responses such as «MAPK signaling pathway» (p = 1.4×10^−2^). The Functional Annotation Clustering tool from DAVID grouped these terms in a single annotation cluster with the best enrichment score (1.36). Nine genes belonging to the «Hematopoietic cell lineage» pathway (p = 6.5×10^−2^) were present in this cluster. As expected in inflammation response, immune and pro-inflammatory responses are activated. These results validate our experimental strategy, particularly the use of OA to induce a reliable inflammation without any infection.

**Table 1:** Functional annotation and enrichment of the 365 genes belonging to cluster Early.

Functional classification			
*Database*	*Term*	*Count*	*P-Value*
Gene Ontology	Immune response	23	6.50E-03
Gene Ontology	Inflammatory response	14	6.90E-03
Gene Ontology	Defense response	18	9.90E-03
Gene Ontology	Cytokine activity	13	1.10E-02
Kegg pathway	MAPK signaling pathway	16	1.40E-02
Gene Ontology	Response to wounding	16	1.90E-02
Gene Ontology	Immune system process	30	2.30E-02
Gene Ontology	Protein kinase cascade	14	3.10E-02
Gene Ontology	Positive regulation of kinase activity	8	3.30E-02
Gene Ontology	Chemokine activity	6	5.00E-02
Gene Ontology	MAP kinase tyrosine/serine/threonine phosphatase activity	3	5.10E-02
Gene Ontology	MAP kinase phosphatase activity	3	5.10E-02
Gene Ontology	Chemokine receptor binding	6	5.60E-02
Kegg pathway	Hematopoietic cell lineage	9	6.50E-02
Gene Ontology	Interleukin-1 receptor binding	3	6.90E-02
Gene Ontology	MAPKKK cascade	7	7.40E-02

Annotation Cluster 1	Enrichment score: 1.36		
*Database*	*Term*	*Count*	*P-Value*
Gene ontology	Immune response	23	6.50E-03
Gene ontology	Inflammatory response	14	6.90E-03
Gene ontology	Defense response	18	9.90E-03
Gene ontology	Cytokine activity	13	1.10E-02
Gene ontology	Response to wounding	16	1.90E-02
Gene ontology	Immune system process	30	2.30E-02
Gene ontology	Response to external stimulus	19	3.30E-02
Gene ontology	Chemokine activity	6	5.00E-02
Gene ontology	Chemokine receptor binding	6	5.60E-02

Most of the genes grouped in the cluster Intermediate were found to be involved in Kegg pathways related to the immune response, notably the spreading of immune cells into inflamed tissues through interaction with vascular endothelial cells, such as «Leukocyte Transendothelial migration» (p = 7.8×10^−3^) or «Adherens junction» (p = 3.5×10^−2^) (table 2). This was further supported by the analysis of Biocarta Pathway, which pointed out several pathways such as «Adhesion Molecules on Lymphocyte» (p = 8.5×10^−3^) and «Monocyte and its Surface Molecules» (p = 1.8×10^−2^). These pathways were clustered together by the Functional Annotation Clustering tool from DAVID in a single cluster with an enrichment score of 1.16. In addition, 5 genes involved in differentiation of T and B lymphocytes cells were found to be involved in the «Hematopoietic cell lineage» pathway (p = 4.2×10^−2^). Surprisingly, genes involved in the spreading of inflammation were grouped together. Usually, this process can not be clearly visualized because of the very short period of time between activation of the inflammatory and immune and anti-inflammatory response.

**Table 2:** Functional annotation and enrichment of the 269 genes belonging to cluster Intermediate.

Functional classification			
*Database*	*Term*	*Count*	*P-Value*
Kegg pathway	Leukocyte transendothelial migration	8	7.80E-03
Biocarta	Adhesion molecules on lymphocyte	4	8.50E-03
Biocarta	Neutrophil and its surface molecules	4	8.50E-03
Biocarta	Monocyte and its surface molecules	4	1.80E-02
Kegg pathway	Adherens junction	6	3.50E-02
Kegg pathway	Hematopoietic cell lineage	7	4.20E-02

Annotation Cluster 1	Enrichment score: 1.16		
*Database*	*Term*	*Count*	*P-Value*
Biocarta	Adhesion molecules on lymphocyte	4	8.50E-03
Biocarta	Neutrophil and its surface molecules	4	8.50E-03
Biocarta	Monocyte and its surface molecules	4	1.80E-02
Gene Ontology	Wound healing	5	9.40E-02

**Figure 5:**
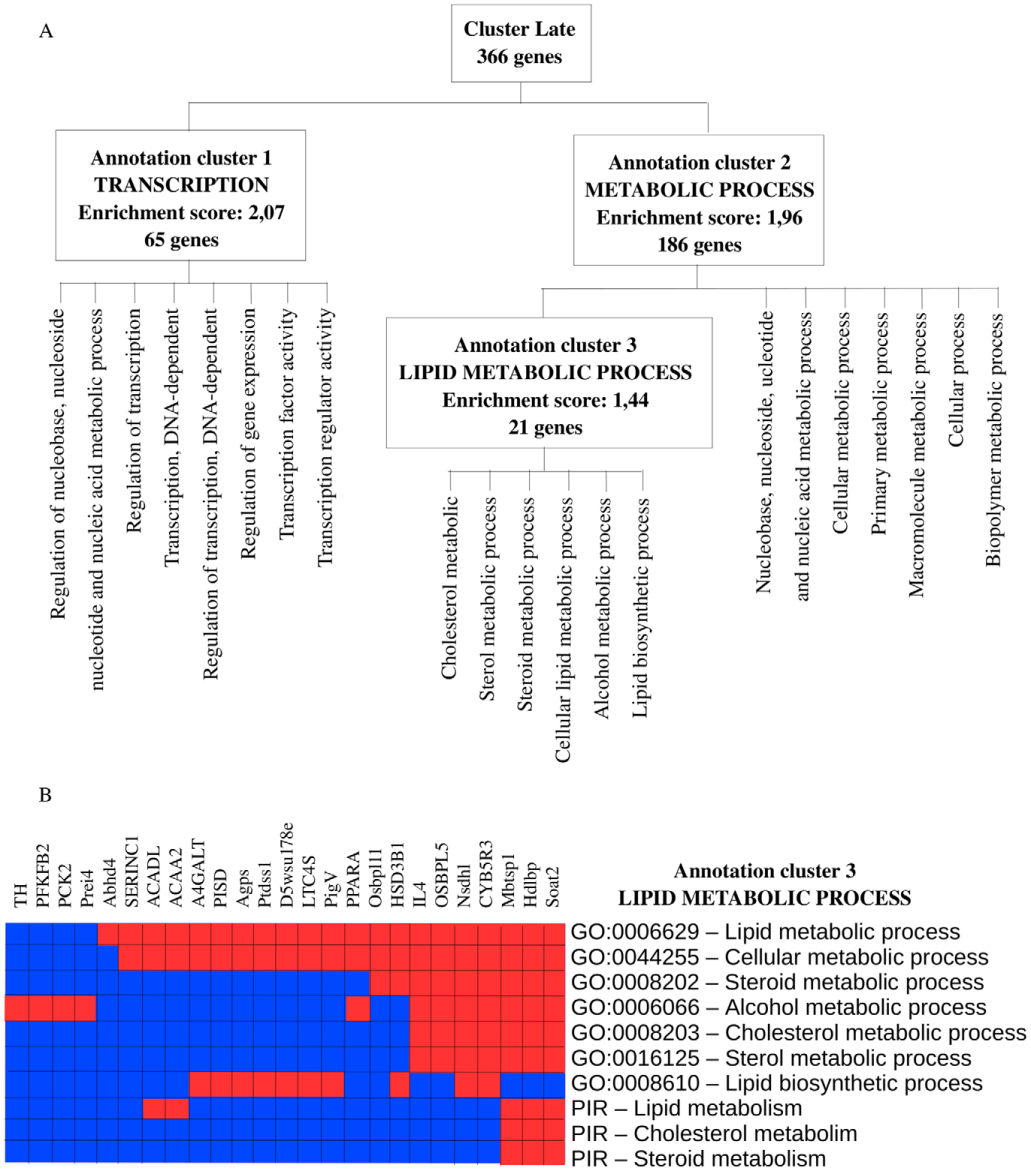
Functional annotation enrichment of the 366 genes belonging to cluster Late. Expression levels of genes belonging to cluster Late increases 3H-4H after OA injection and reach a peak after 18H to 24H incubation. A- Distribution of the 366 genes belonging to Cluster Late into the 3 main annotation clusters identified by the functional annotation clustering tool from DAVID: Transcription, Metabolic process and Lipid metabolic process. B- 21 genes in cluster Late belong to the lipid metabolic process. These genes can be assigned to one or several of 7 GO categories related to Lipid metabolism. Three of them also belong to 3 PIR categories (Protein Information resources) related to lipid metabolism. Red means the gene belong to the pathway and Blue means that the gene does not belong to the pathway.

Functional annotation analysis of genes belonging to the cluster Late showed an over-representation of GO terms related to transcription, metabolism and in particular lipid metabolism (figure 5 and supplementary table S1). The Functional Annotation Clustering tool from DAVID identified 3 groups of genes with the highest enrichment scores. The first one (enrichment score  = 2.07) showed an over-representation of GO terms related to «Transcription» (p = 1.4×10^−3^), such as «Regulation of transcription» (p = 1.1×10^−3^) or «Regulation of nucleobase, nucleoside, nucleotide and nucleic acid metabolic process» (p = 7.7×10^−4^). Most of the GO terms grouped in the second annotation cluster (enrichment score  = 1.96) were found to be involved in metabolism, such as «Metabolic process» (p = 2.2×10^−3^) or «Cellular metabolic process» (p = 2.3×10^−3^). The third annotation cluster (enrichment score  = 1.44) included GO terms related to lipid metabolism such as «Cholesterol metabolic process» (p = 9.3×10^−3^) or «Cellular lipid metabolic process» (p = 5.2×10^−2^). Increased expression level of genes involved in transcription is a consequence of the metabolism which is highly perturbed. Multiple alterations in lipid and lipoprotein metabolism have been previously described associated with infection and inflammation [16]. Here, we observed that disturbance of genes expression levels related to lipid metabolism is associated only with inflammation.

We also considered genes of interest for clinical studies such as Smad7 [17]. In our study, Smad7 belongs to the set of significantly differentially expressed genes across time and it belongs to cluster Late. Its expression level increased between 3 hours and 4 hours after OA injection to reach a maximum expression level 24 hours after injection. We also considered the angiotensin-converting enzyme gene (Ace) [18]–[19]. In our study, Ace expression level did not significantly change over time. Finally, we considered transcription of genes involved in surfactant lipid biosynthesis and surfactant-associated proteins. In a previous study, authors quantified expression levels of 5 genes involved in the surfactant lipid biosynthesis and 5 genes involved in the surfactant-associated protein biosynthesis [20]. Expression levels of these genes decreased significantly in mice developing ALI. Out of these 10 genes, 4 are present on MUSV29K: Slc34a, Fasn, Sftpd and Napsa. In our study, Slc34a, involved in surfactant lipid biosynthesis, is the only gene whose expression level changed significantly over time. It belongs to the cluster Late and its expression level increased over time after OA injection to reach a peak at 24 hours incubation.

Overall, our gene expression analysis revealed marked changes in pathways involved in the immune and inflammatory responses, and also lipid metabolism and transcription. First, the pro-inflammatory phase is activated with high expression level of genes involved in inflammation and immune response. Then, inflammation is spreading into inflamed tissues. And, later, transcription is activated and lipid metabolism seems highly disturbed.

## Discussion

### Data summary

In this study, we have searched for genes and physiological pathways potentially involved in ALI. To this purpose, we analyzed differentially expressed genes in lung from C57BL/6J mice at different stages of inflammation. We found that injection of OA deeply alters gene expression. In particular, we identified 3 clusters of genes specifically highly expressed either at early time-points (early-inflammatory stage), at late time-points (late-inflammatory stage), or at the time-points when the shift between early- and late-inflammatory stages occurs. In order to identify genes whose changes in expression levels are associated with lung inflammation, we performed a three class SAM procedure with a stringent false discovery rate of 5%. The procedure was conducted on the whole dataset. Because the technical procedure may affect the gene expression level, we adjusted the measurements of mice treated with OA to that treated with physiological serum. Thus, we identified 1000 genes differentially expressed between OA mice and physiological serum mice.

### Limits of the OA model

In this study, our first aim was to define a model of lung inflammation in mouse using OA. Despite its widespread use, the OA-induced ALI model has not been standardized. One to 2 ml of OA generates ALI when injected as a single bolus directly into the pulmonary circulation to an animal weighing 25 Kg [10]. However, both smaller and larger doses have also been used. At other times, the injection has been given in fractions, after sonication in saline, after OA dissolution in ethanol, or as continuous infusion. Each of these variations may alter the severity or extensiveness of the injury, resulting in different physiopathologic consequences. For example, Zhou *et al.* determined that an intravenous dose of 0.15 µL/g body weight OA in mice resulted in an approximate 24-hour survival rate of 75% [12]. Sixty minutes after OA injection, they observed severe alveolar damage with the development of alveolar edema, increased-permeability and abnormalities in oxygenation. Using doses of OA that were up to approximately 1.5 times the dose used by Zhou *et al.*, Ulrich *et al.* reported similar histopathological findings 1 hour after OA injection in mice [13]. A dose-related difference in mortality was observed in animals treated with 0.2 and 0.4 µL/g of OA administered through the tail vein, respectively. The higher dose was associated with 100% mortality, 30 to 40 minutes following OA administration.

Previous studies showed that a time-course ranging from 40 to 90 minutes was required to detect an inflammatory response [12]. The length of the incubation period required to detect a pro-inflammatory profile (expression levels of pro- and anti-inflammatory cytokine or lung histology) depended on the OA dosage. As mechanical ventilation induces by itself an inflammation [21], one can hypothesize that models using mechanical ventilation increased the inflammatory response. Given the contradictory results previously published regarding the kinetics of inflammation in mice, we quantified mRNA levels of pro- and anti-inflammatory cytokines before performing high-throughput gene expression analysis. Cytokines play a critical role as signaling molecules that initiate, amplify, and perpetuate inflammatory responses on a local and systemic basis.

### Timing of inflammation

We quantified expression levels of 4 cytokines 1 hour, 1.5 hours, 3 hours, 4 hours, 18 hours and 24 hours after OA incubation. Tnfα, Il6, Il10 and Il4 are commonly used to specifically study pro- and anti-inflammatory phases (figure 1) [22].

Tnfα is an early response cytokine. It plays an important role in immunity and inflammation, in the control of cell proliferation, differentiation and programmed cell death [23]. We found that, in our model without infection, Tnfα is temporarily highly expressed 60 to 90 minutes after OA injection (figure 1 B). This corresponds to the pro-inflammatory response.

Il6 is produced by a wide range of cells including monocytes/macrophages, endothelial cells, fibroblasts, and smooth muscle cells in response to stimulation by endotoxin [24]. Raised levels of Il6 have been described in a number of acute conditions such as burns, major surgery and sepsis [25]. Il6 is a ubiquitous cytokine playing a role on pro- and anti-inflammation. Several experimental models suggest a protective role for Il6 against inflammation [26]. Pro-inflammatory effects of Il6 have been shown in several tumor cell lines [27]. Il6 is also involved in the spreading of pro-inflammatory cells [28]. In our study, expression level of Il6 is maximum 3 hours after OA injection, interconnecting the pro- and anti-inflammatory phases (figure 1 C).

Il10, an anti-inflammatory cytokine [29], inhibits the release of pro-inflammatory cytokines such as Tnfα from monocytes/macrophages, thus preventing subsequent tissue damage [30]. The role of Il10 is essential to counter balance the pro-inflammatory response. In a previous study, Park *et al.* found a disproportionate anti-inflammatory response to the early pro-inflammatory response, which leads to ARDS [31]. In our study, Il10 expression increased from 3 hours and peaked 4 hours after OA injection which corresponds to the anti-inflammatory response to Tnfα (figure 1 D).

Il4 is also an anti-inflammatory cytokine which down-regulates local levels of pro-inflammatory cytokines and chemokines [32]. We found that its production was simultaneous with Il10 expression peak. This finding confirms the timing of the anti-inflammatory phase (figure 1 E).

The quantification of expression levels of 4 pro- and anti-inflammatory cytokines by real-time PCR allowed us to identify two phases during inflammation. We identified the pro-inflammatory phase based on the high expression level of Tnfα 1.5 hours after OA injection. Later, 3 to 4 hours after OA injection, anti-inflammatory cytokines Il10 and Il4 are highly expressed. Meantime, Il6, which has pro- and anti-inflammatory activities, is highly transcribed 3 hours after OA injection. Given the contradictory results previously published, this preliminary step was essential to timely identify pro- and anti-inflammatory phases in our model and then perform large-scale transcriptional study considering each phase of inflammation described.

### An early inflammatory and immune response

Once our mouse model of lung inflammation settled, we identified molecular pathways associated with lung injury.

**Figure 6:**
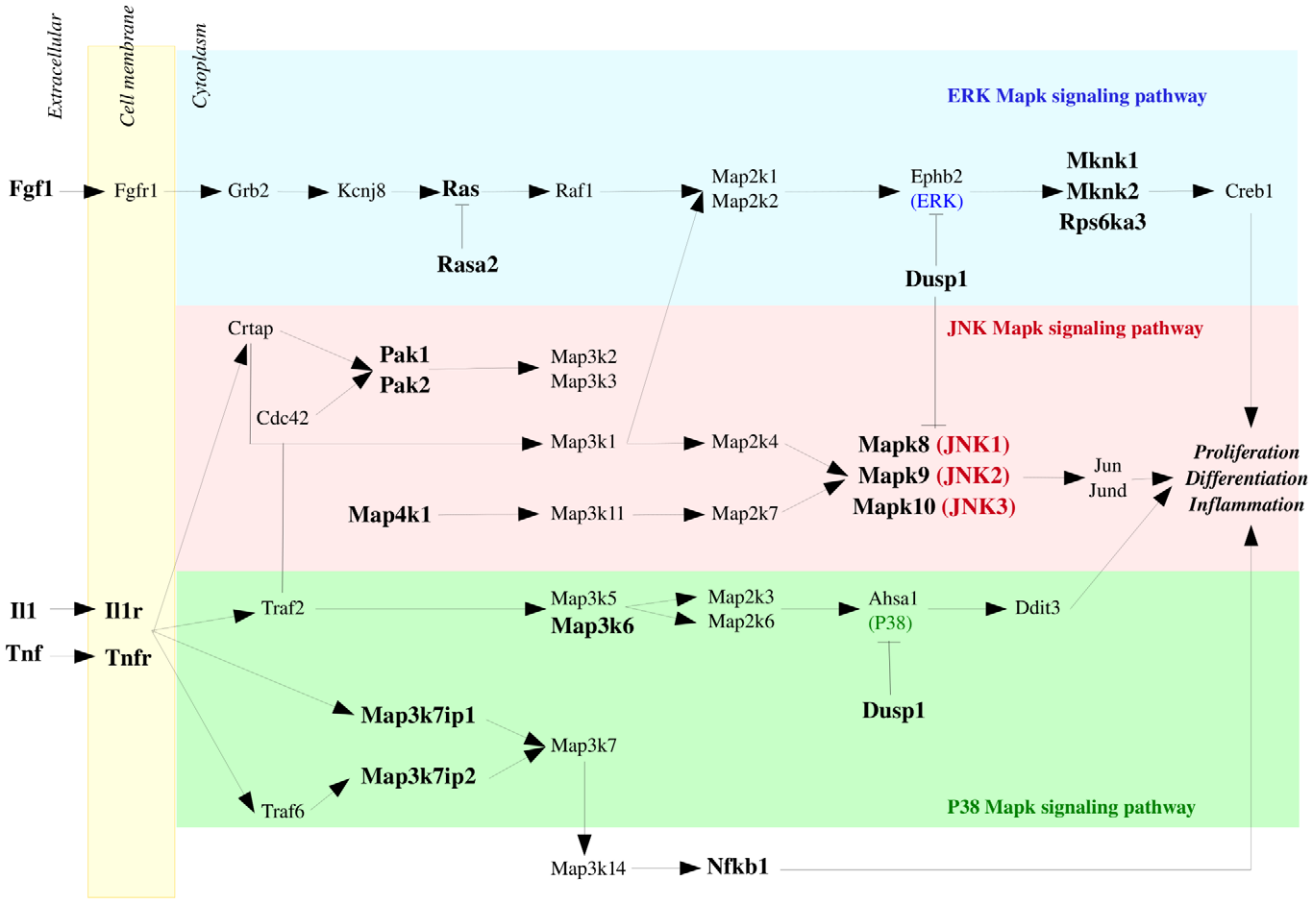
MAPKinase Signaling Pathway. The 3 major groupings of mitogen-activated protein kinase (MAPkinase) pathways are activated rapidly after OA injection. Genes belonging to the extracellular signal-related kinases (ERK) (blue), the Jun amino-terminal kinases (pink) and the p38 proteins (green) are up-regulated early after OA injection. These genes are represented in bold.

Injection of OA rapidly affects the expression of genes involved in the defense response such as inflammatory and immune responses. Expression level of numerous chemokines such as Cxcl5, Ccl22, Ccl4, Ccl3, Ccl6 or Ccl25 increased rapidly and allowed recruitment of specific leukocyte subpopulations to sites of tissue damage (see supplementary data file S4) [33]. The expression of pro-inflammatory cytokines and chemokines has been previously observed in transcriptional studies of infection in model organisms. Calvano *et al.* showed, in human blood leukocytes, an over-expression of pro-inflammatory chemokines 2 hours to 4 hours after endotoxin administration [34]. The activation of cytokines following inflammation has also been observed in lung by Chinnaiyan *et al.* in a transcriptional study of systemic inflammation in a cecal ligation/puncture model of sepsis in rat [35]. Also, the MAPK signaling pathway, involved in cell proliferation, differentiation and inflammation is activated (see supplementary data file S4) (figure 6). Three regulated groups of MAPK are activated: the extracellular signal-related kinases (ERK) with genes such as Fgf1, Rasa2, Ras, Dusp1, Mknk1, Mknk2 or Rps6ka3; the Jun amino-terminal kinases (Mapk8, Mapk9, Mapk10) with Il1, Map4k1, Pak1, Pak2 and Dusp1; and the p38 proteins with Il1, Map3k7ip1, Map3k7ip2, Dusp1 and Map3k6. Il1b is known to participate in ARDS pathogenesis [36]. The MAPK cascade pathways are critical for the transmission of activated cell surface receptor signals to invoke multiple regulated intracellular processes. We also noticed over-expression of Il15ra which blocks Il15, an inhibitor of Tnfα [37]. Activation of inflammatory and immune responses early after OA injection validates our inflammation model in mouse [23].

### An intermediate spreading of inflammation

The transition phase at 3 hours –4 hours which followed the pro-inflammatory phase corresponds to the spreading of inflammation. Among genes whose expression level reaches a maximum at this time-point, we found transmembrane adhesion molecules (Cd44, Pecam1 and Icam1) as well as endothelial cell adhesion molecule (CAM) (Cd34, Icam1, Glycam1 and Pecam1) (see supplementary data file S4) [37]–[38]. It has been previously shown that CAM adhesion molecules are strongly involved in the inflammatory process by modulating the leukocyte trafficking [38]–[39]. B and T cell lymphocytes interact with a variety of cells as part of their immune function allowing inflammatory signals to circulate between organs. The interaction of lymphocytes with other cell types like vascular endothelial cells requires transmembrane adhesion molecules. Each of these lymphocyte adhesion molecules interacts with specific ligands expressed on cells like endothelial cells to moderate adhesion between cells. Cd44 is an adhesion molecule with several roles, including interaction of leukocytes with endothelial cells in response to inflammation [40]. Pecam1 mediates the spreading of monocytes and other immune cells into inflamed tissues through interaction with vascular endothelial cells [41]. Expression of intercellular adhesion molecule-1 (Icam1) is increased by inflammatory signals, leading to higher adhesion and permeation of lymphocytes into inflamed tissues. Icam1 is an inducible protein expressed on the surface of endothelial cells. Under physiological conditions, Icam1 is not constitutively expressed, or is expressed at low levels in most tissues. In inflammatory states, it has been shown that expression level of Icam1 is up-regulated by Tnfa [42]. It has been shown that increased expression of Icam1 protects lung against injury by neutralizing antibodies or targeted gene deletion during bacterial sepsis, acute pancreatitis and trauma [43]. As Icam1 deficiency interferes with neutrophil recruitment, these results support the concept of a therapeutic strategy directed against neutrophil migration and activation. During inflammation, leukocytes bind to endothelial CAM and then migrate across the vascular endothelium.

There is a clear link between blood and inflammation as blood carries the inflammation throughout the entire organism. We found, in cluster Intermediate, 9 genes such as Cd34, Cd44, Il7r, Csf1 and Cd38 leading to differentiation of blood cells (see supplementary data file S4). Blood-cell development progresses from an hematopoietic stem cell which can differentiate into several lineages such as T lymphocytes, B lymphocytes, NK cells, erythrocytes or platelets. Cells undergoing this differentiation process express a stage- and lineage-specific set of surface markers. Therefore cellular stages are identified by the specific expression patterns of these genes. Cd34 is an adhesion molecule with a role in early hematopoiesis by mediating the attachment of stem cells to the bone marrow extracellular matrix or directly to stromal cells [44]. Cd44 is involved in lymphocyte activation [40]. Il7r is the receptor of Il7 which is involved in the regulation of lymphopoiesis. Response of cells to Il7 is dependent on the presence of the Il7r [45]. Csf1 induces cells of the monocyte/macrophage lineage [46].

We were able to visualize the spreading of inflammation as many genes involved in leukocyte transendothelial migration and CAM clustered together. Spreading of inflammation is part of the pro-inflammatory response to inflammation. We observed that high expression level of genes involved in the spreading of inflammation signal follows the high expression level of genes involved in inflammation and immune processes. This is probably due to the fact that vascular endothelium is the target of pro-inflammatory cytokines. Once the vascular endothelium activated, genes involved in cell adhesion are expressed and leukocytes are transferred from circulating blood to the site of inflammation.

### A late disruption of lipid metabolism

In our study, we found a late disruption of gene expression level related to lipid metabolism. Indeed, among numerous genes whose expression level increases at 3 to 4 hours and reach a peak at 18 hours–24 hours, 21 belong to the lipid metabolic process (figure 5 and see supplementary data file S4). Previous studies showed lipid metabolism disturbance soon after inflammation started. Hardardottir *et al.* have proposed that the changes in lipid and lipoprotein metabolism that occur during the host response to infection/inflammation include anti-infective and anti-inflammatory effects that contribute to the host defense [47]. Infection and inflammation are accompanied by cytokine-induced alterations in lipid and lipoprotein metabolism [48]. As expression level of numerous genes involved in lipid metabolism increased 3 to 4 hours after OA injection and are highly over-expressed 24 hours after OA injection, we believe that lipid metabolism disruption is related to the highly transcribed pro-inflammatory cytokines such as Tnfα and Il1. Indeed, we observed that expression level of Tnfα is maximum 1.5 hours after OA administration (figure 1 B) and Il1, which belongs to cluster Early, is highly expressed 1 hour–1.5 hours after OA administration (figure 4). Il6 is highly expressed 3 hours after OA injection. Moreover, it has been previously shown that serum triglyceride levels, serum total cholesterol and LDL levels increased as a consequence of the highly transcribed pro-inflammatory cytokines [47], [49]. Simultaneously, during inflammation, there is a marked decrease in serum levels of HDL [50]. We do not believe that high expression level of genes involved in lipid metabolism is a direct consequence of OA itself. Indeed, even if OA belongs to the fatty acid family, several studies have shown that late disruption of the lipid metabolism is associated with inflammation. Worgall, for example, showed that expression of defective cystic fibrosis transmembrane conductance regulator (CFTR), the cause for cystic fibrosis, is associated with an inflammatory state and affects fatty acid and cholesterol metabolism which is associated, in great majority of patients, with decreased linoleic acid level and increased myristic, palmitoleic, stearic and oleic fatty acids levels [51]. Recently, there has been much interest in using Statin for sepsis [52]. Statin, which has lipid-lowering properties, reduce total cholesterol, LDL and triglyceride levels [53]. These previous works showed that triggers of inflammation which are not fatty acids led to disruption of lipid metabolism. Feingold *et al.* observed that the hypertriglyceridemic effect of LPS and cytokines is rapid, occurring 2 hours after administration and is sustained for at least 24 hours [16]. Channaiyan *et al*. showed, in a transcriptional study of systemic inflammation in a cecal ligation/puncture model (CLP) of sepsis in rat, that several genes involved in lipid metabolism are up-regulated in lung 6H after CLP [35]. In our study, the timing of the cytokine effect on genes related to lipid metabolism is similar. We showed here that lipid metabolism disturbance is a consequence of inflammation.

Most of the changes in genes related to lipid metabolism that are induced by inflammation are attributable to changes in gene transcription. The expression level of 189 genes involved in at least one out of the 25 GO categories belonging to the transcriptional process are part of the annotation cluster 1 identified by DAVID. They slowly increased 3 hours to 4 hours after OA injection to reach a maximum expression level after 18 hours to 24 hours OA incubation (supplementary table S1). This is probably the consequence of the high expression level of many pro-inflammatory cytokines identified in cluster Early (figure 5). In the promoter region of Il6 and Il1, transcription factors Nfkb1 and Cebpb (NF-IL6) are involved in the expression of many inducible cellular genes that encode cytokines, immunoregulatory receptors, and acute phase proteins [50]. Because many genes involved in regulating immune response and acute phase reaction contain both Cebpb and Nfkb1 sites, it is highly possible that cooperative interactions between Cebpb and Nfkb1 play an important role in the expression of cytokines. For many positive acute phase proteins, transcription activities of their corresponding genes increase during inflammation, reaching a maximum level between 18 hours and 36 hours after inducing an acute inflammation [54]. Our results confirm these findings. Indeed, pro- and anti-inflammatory cytokines highly expressed respectively at early and late stages of the inflammation process induce the transcription of many other genes later after OA injection. Also, Calvano *et al.* showed in their microarray study in 2005 that, in human blood leukocytes, 4 hours to 6 hours after endotoxin injection, the expression of numerous transcription factors was increased. Among them, the expression of several members of the nuclear factor kappa/relA family of transcription factors (Nfkb1, Nfkb2, Rela, and Relb) reached their zenith [34].

We expected a massive release of inflammatory mediators soon after OA injection followed by the release of anti-inflammatory mediators [55]. This sequence of events has been previously described by authors studying sepsis [56]. The response to inflammation we observed and which lasted over 24 hours has been previously described associated with infection [47]. Observing these responses associated with only inflammation justifies the need to fully describe gene expression profiles during inflammation and therefore, it will become possible for other research groups to identify specific responses to infections during sepsis.

### Potential targets for therapy

By looking at how changes in gene expression occur through time during lung inflammation, we could confirm or invalidate targets previously identified for therapy.

Among these candidates, Smad7 has been identified [57]. Transient gene transfer and expression of Smad7, introduced by recombinant human type 5 adenovirus vector into the lungs, prevented pulmonary fibrosis induced by bleomycin in mice [57]. Smad7 is an intracellular antagonist of Tgfb signaling [57]. It inhibits Tgfb-induced transcriptional response. Moreover, it is thought that prolonged overproduction of Tgfb induced by repeated chemical or biological injury leads to the accumulation of pathological amounts of extra-cellular matrix in the lung tissue, which is followed by functional deterioration [17]. We believe Smad7 is a good candidate for gene therapy because it belongs to the set of significantly differentially expressed genes across time and its expression level increased 3 to 4 hours after OA injection to reach a maximum expression level 24 hours after injection.

We also considered the angiotensin-converting enzyme gene (Ace) which can predict susceptibility and outcome in ARDS. Circulating Ace derives largely from the pulmonary endothelium. Its release in circulation is affected by extensive endothelial damage [18]. Septic ARDS patients have markedly decreased serum Ace levels compared to those of non-septic ARDS patients [19]. In our non-septic model of ALI, Ace expression level did not significantly change over time.

Finally, we considered transcription of surfactant lipid and surfactant-associated proteins. During ALI in mice, epithelial cell injury leads to reduced surfactant biosynthesis. It has been previously shown that maintenance of surfactant-associated protein B (Sftpb) transcript levels is necessary for over-expression of mouse transforming growth factor α (Tgfa) involved in protection against nikel-induced lung injury [20]. Out of the 10 candidate genes previously identified, 4 are present on MUSV29K: Slc34a, Fasn, Sftpd and Napsa. In our study, Slc34a, involved in surfactant lipid biosynthesis, is the only gene whose expression level changed significantly over time. It belongs to the cluster Late and its expression level increased over time after OA injection to reach a peak at 24 hours incubation.

Our wide-scale transcriptional study identified pathways and specific genes involved in response to inflammation. We found several potential candidates for gene therapy such as Smad7. Since there has been great progress in delivering genes to the lungs over the past decade, gene therapy is expected to become a new tool in addition to classical approaches for treating inherited and acquired diseases including ARDS in the near future.

### Conclusion

In this study, we pictured gene expression profiles during inflammation according to the time-course. Overall, our microarray analysis gives a global overview of critical events occurring during lung inflammation. We have first confirmed that gene-expression profiling discriminates between pro- and anti-inflammatory phases during lung inflammation. The analysis of gene functional annotation reveals several major features. First, it visualizes at transcription level the pro-inflammatory stage characterized by the release of immune response and activation of inflammatory mechanisms. We observed that these pathways are activated soon after the initiation of lung injury. Then the Intermediate cluster revealed that the immune cells migrate into inflamed tissues through interaction with vascular endothelial cells. Finally, at late stage of the inflammation, metabolism is deeply disturbed. We also showed here that inflammation is associated with marked changes in lipid and lipoprotein metabolism suggesting that lipoproteins seem to participate in innate immunity.

This inflammation model in mouse is the first step toward the identification of the molecular mechanisms involved in the infection process at different steps of inflammation. From now on, when studying the impact of an infection at the transcriptional level, we will be able to distinguish between the gene expression level variations due to the pathogen itself and the ones resulting specifically from the inflammation associated with infection. In our study, by identifying numerous common pathways, we showed that the inflammation alone which is a component of infection has to be considered separately from infection.

Furthermore, it is reasonable to think that the impact of a pathogen on an inflamed organ depends on the pre-existing inflammation. Inoculation of an infectious agent has to take into account timing of the pre-existing inflammation (pro- or anti-inflammatory stage). We reached our first objective of characterizing inflammation across a time-course. Next, we will induce an infection in mouse undergoing inflammation at different time-points (pro- vs. anti- inflammatory phases) using group B streptococcus.

## Materials and Methods

### Experimental groups

**Figure 7:**
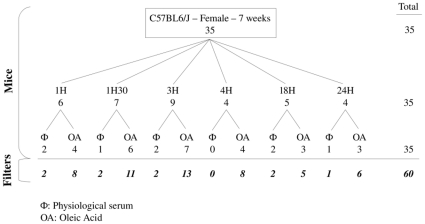
Experimental design of the experiment. We studied 35 mice C57BL6/J, female, 7 weeks old. We divided these mice into 6 groups based on the incubation time (1H, 1H30, 3H, 4H, 18H, and 24H). In each group, mice are divided into 2 subgroups depending on the injection: OA or physiological serum. 25 samples have been hybridized on 2 microarrays consisting in technical replicates and 10 samples have been hybridized once. We performed 60 hybridizations.

Wild-type female C57Bl/6J mice, 7 weeks old, were obtained from Charles River and housed in a specific pathogen-free animal facility. We studied 35 mice divided into 6 groups (figure 7). Each group included one or two mice administered with physiological serum and 3 to 7 mice administered with OA, which correspond to biological replicates. Each group was identified according to the incubation time: 1 hour, 1.5 hours, 3 hours, 4 hours, 18 hours and 24 hours. All experimental procedures involving animals were approved by the veterinary office of the Ministry of Agriculture, France (authorization number: 13–27).

### Injections

Female C57Bl/6J mice were anesthetized with 5% Xylazine–20% Ketamine (0,1 ml/10 g). We injected 20 µl of physiological serum in the control mouse at each time-point and 20 µl of OA (1,2 µl/g body weight, sigma #27728-1L-R) in the other 27 mice of our study. Administration of physiological serum and OA was done through the tail vein with a 0.3-ml insulin syringe (BD #320837).

### Sacrifice and organ removal

Each group of mice was sacrificed 1 hour, 1.5 hours, 3 hours, 4 hours, 18 hours or 24 hours after injection of OA or physiological serum by exanguination through the eye vein. For each mouse, we collected the smallest lung for RNA extraction in 4 ml Trizol. The lung was immediately fractionated using a polytron (T8 Ultra-Turrax, IKA WERKE #3420000) and kept at −20°C.

### Lung histology

The lung was fixed in 4% Paraformaldehyde and paraffin embedded. Five µm sections were cut and stained with Harri's hematoxylin and eosin (H&E) for morphological examination. Lung inflammation was assessed by studying the following morphological criteria: alveolar/interstitial edema, presence of fibrin, alveolar/interstitial neutrophils. All slides were coded and evaluated in a blinded fashion to prevent bias.

### RNA extraction

Total RNA from lung was extracted using TRIzol reagent (Invitrogen, #15596-026). The quality of RNA was confirmed on a 1% denaturing agarose gel, and the concentration of RNA was determined by reading absorbance at 260/280 nm.

### Real-Time PCR

Real-time RT-PCR amplification was performed using primer sets for one control gene: Actb (5′-ACTCTTCCAGCCTTCCTTC-3′ and 5′-ATCTCCTTCTGCATCCTGTC-3′), two pro-inflammatory genes: Tnfα (5′-CCCTCACACTCAGATCATCTTCT-3′ and 5′-GCTACGACGTGGGCTACAG-3′) and Il6 (5′-TAGTCCTTCCTACCCAATTTCC-3′ and 5′-TTGGTCCTTAGCCACTCCTTC-3′), and two anti-inflammatory genes: Il10 (5′-GCTCTTACTGACTGGCATGAG-3′ and 5′-CGCAGCTCTAGGAGCATGTG-3′) and Il4 (5′-TGGATCTGGGAGCATCAAGGT-3′ and 5′-TGGAAGTGCGGATGTAGTCAG-3′). cDNA was synthesized using 5 µg of RNA, 1 mM dNTP, and1 µg dt25 primer. After 5minutes at 65°C, 10 mM DTT, 40 U RNAaseIN (Promega,#N261B) and 400U of Superscript II transcriptase (Invitrogen, #18064-014) were added. This mix has been maintained 2 hours at 42°C and 15 minutes at 70°C. 1 µl of RNAaseH (Invitrogen,#18021-014) has then been added and the samples have been kept at 37°C for 20 minutes. 1 µl of the RT product was then amplified for 2minutes at 50°C, 10minutes at 95°C followed by 40 cycles (15 sec at 95°C, 30 sec at 60°C, 30 sec at 72°C) followed by one cycle of 72°C for 10minutes and 95°C for 15 sec. This was done in a 25 µl reaction mix containing 1X Power SYBR Green PCR Master Mix (Applied Biosystem, #4367659) and 25pM of each primer using the ABI PRISM 7000 sequence Detection System (Applied Biosystem).

Relative expression of the RT-PCR products was determined using the comparative CT method based on the mathematical model from M.W. Pfaffl [58]. Relative expression values for each pro-inflammatory and anti-inflammatory gene were expressed as a ratio of pro/anti-inflammatory gene expression level after OA injection to the same pro/anti-inflammatory gene expression level after physiological serum at the same time-point. Each sample was run in triplicate. At least 2 negative controls without template or with lung RNA were systematically conducted with each amplification.

### Microarray design

cDNA microarrays were designed and prepared as described by Puthier *et al.* [59]. The microarrays used in this study, MUSV29K, contain 7771 sequences. The following cDNA libraries were used: the NIA Mouse 15K cDNA clone set, 2NbMT (thymus), NbMLN (lymph node), and 3NbMS (spleen). Detailed descriptions of these cDNA libraries are available at the UniGene database website (2NbMT: Lib.544, 3NbMS: Lib. 553, NbMLN: Lib.567, NIA 15K: Lib. 8622) [60]. All of the libraries were cloned into pT3T7D-Pac vector, except for the NIA 15K Mouse cDNA clone set, which was cloned into pSPORT1 vector. The NIA 15K Mouse cDNA clone set is a re-arrayed and resequenced set of 15 000 bacterial clones derived from 11 embryo cDNA libraries [61]. 2NbMT, NbMLN and 3NbMS are sequenced I.M.A.G.E libraries. These libraries contain 84127 clones. We used the Microarrays Quality Control tool to select clones matching a single region on the mouse genome and for which at least one 3′ EST and one 5′EST have been identified [62]. We selected 7771 bacterial clones matching 6622 mouse genes. 73% (4833) of the genes are represented by a single cDNA clone and about 27% (1789) of the genes included in this gene set are represented by two or more different cDNA clones, providing internal controls to assess the reproducibility of gene expression measurements. We also added 8 positive controls (poly-A LBP2S and Cot1, a mix of DNA fragments containing repeated sequences) and 2205 negative controls (empty spots and CG03, an *Arabidopsis thaliana* cDNA sequence). In summary, the MUSV29K mouse microarray we designed contains 9984 spots, identifying 6622 mouse genes.

### Microarray preparation

PCR amplifications were performed in 96-well microliters plates using the following primers: 5′-CCAGTCACGACGTTGTAAAACGAC-3′ and 5′-GTGTGGAATTGTGAGCGGATAACAA-3′. The reactions were performed as described in Diehl *et al.* [63] by transferring few *Escherichia coli* from a growth culture with a plastic 96pins replicator (Proteigene, #X5055) to 100 µl of PCR mix pH 8,5 containing 1,5 mM MgCl2, 1 M Betain, 375 µM dATP, dTTP, dGTP, dCTP, and 5 U of GoTaq DNA polymerase (Promega, #M3171). The plates were incubated for 6 min at 94°C, before 38 cycles of 94°C for 30 seconds, 65°C for 45 seconds and 72°C for 3.5 minutes, followed by a final elongation phase at 72°C for 10 minutes. Amplification products were not quantified, but their quality was systematically checked on 1% agarose gels. 11,03% of the bacterial clones were estimated to be non-amplified and 4,38% showed multiple bands after PCR amplification. Unpurified PCR products were then transferred to 384-well microplates using the TECAN Genesis workstation 150, before being evaporated. Each well was filled with 30 µl of distilled water, and its content spotted onto nylon membranes (Hybond-N+; Amersham Bioscience). This step was conducted using a MicroGrid-II arrayer (Apogent Discoveries) equipped with a 64-pin biorobotics printhead. Microarrays were hybridized with χdATP 33P-labelled probe, whose oligonucleotide sequence is common to all spotted PCR product (LBP2S 5′-TCACACAGGAAACAGCTATGAC-3′). It showed uniform signal intensities across individual membranes [64].

### Microarray data acquisition and analysis

10 mRNA samples extracted from 10 mice were run on a single microarray. In addition, 25 samples were run on two microarrays, and were considered as technical replicates. This corresponds to a total of 60 microarrays. All microarray procedures were done at our microarray core facility [65]. CDNA were designed and prepared as described in Puthier *et al.* [59], using 5 µg of total RNA in the presence of α-dCTP 33P.

After image acquisition, data were processed as described in figure 3. Hybridization signals were quantified using the Bzscan2 software [66]. All images were carefully inspected and spots with overestimated intensities due to neighborhood effects were manually excluded. We used the NylonArray library for R locally developed by A. Bergon and D. Puthier [67]. This package contains functions to perform diagnosis and normalization of nylon microarray data. For each microarray, we obtained two datasets: the first was obtained by hybridizing the microarray with a probe, whose oligonucleotide sequence is common to all spotted PCR product (vector) and the second was obtained by hybridizing the microarray with lung cDNA (sample). The sample datasets were corrected for neighborhood effects and local background as described by F. Lopez *et al.* [66]. Quantile normalization was applied to sample data to correct for global intensity and dispersion.

Microarray data were statistically analyzed using the TIGR MeV (MultiExperiment Viewer) V4.3.01 software [68]. SAM (Significant Analysis of Microarrays) procedure was applied to look for time specific variation in gene expression in the dataset [14]. A each time-point, mean value of gene expressions in control samples (physiological serum injection) were subtracted from gene expression in OA samples. Hierarchical clustering (average linkage clustering metrics and Pearson correlation for the distance) was applied to the dataset (samples and genes) using the Cluster software and results were visualized with the Treeview software [15]. We identified biological annotation for the clusters using DAVID [69] and interaction networks using the Kegg pathways [70]. We applied a Bonferroni correction to account for multiple tests performed. To interpret our data, we used two functionalities in DAVID: the «Functional annotation» tool and the «Functional annotation clustering» tool. The first one associated gene ID with a biological term which belongs to one out of the 40 annotation categories available in DAVID, including GO terms, protein-protein interactions, protein functional domains, diseases associations and sequence feature. This extended annotation coverage increases the analytic power by allowing investigators to analyze their genes from many different biological aspects in a single space. DAVID functional annotation clustering measured relationships among the annotation terms based on the share of common genes. This type of grouping of functional annotations was able to give a more insightful view of the relationships between annotation categories and terms compared with the traditional linear list of enriched terms, as highly related/redundant annotation terms may be dispersed among hundreds of other terms. Each cluster of functional annotation was associated with an enrichment score, which depended on the distribution of the enrichment (p-values) of each member. A good enrichment score was obtained when most of members had good p-values. This score is a relative score instead of a statistical probability with a minimum and a maximum value. This means that enrichment scores could be considered only together, by comparing them.

All data are MIAME compliant and have been submitted to the GEO database (http://www.ncbi.nlm.nih.gov/geo/; accession number GSE18712).

## Supporting Information

Data file S1 Comparison of FDR5% and FDR0% to identify significantly differentially expressed genes. In our study, we applied a false discovery rate of 5%, which yielded to a set of 1000 genes significantly differentially expressed across the time­course of our experiment. By filtering our data based on q<0.01 and FDR 5%, we end up with 121 genes significantly differentially expressed in our experiment. By applying a false discovery rate of 0%, we identify 121 genes significantly differentially expressed across the time­course and identical to the ones identified with FDR 5% and q<0.01.
(0.14 MB PDF)

Data file S2 One thousand genes have been identified by SAM with FDR 5%. Each gene is associated with its expression values in OA samples. Each expression value has been normalized by quantile and adjusted with its physiological serum value at the same time point.
(0.40 MB TXT)

Data file S3 Identification of clusters of samples using hierarchical clustering. Prior to partition samples ( = microarrays) into 3 classes (early, intermediate and late), we performed a hierarchical clustering of the samples in TMEV V4.3.01. using average linkage clustering metrics and Pearson correlation for the distance. This analysis partitioned the samples into 3 groups: early (1H­1H30), intermediate (3H­4H) and late (18H­24H).
(0.14 MB PDF)

Data file S4 Sample of interesting genes differentially expressed across the time-course. For each cluster, a set of genes mentioned in the manuscript is listed associated with their average expression level at each time-point.
(0.00 MB TXT)

Table S1 Functional annotation and enrichment of the 366 genes belonging to cluster Late. We identified biological annotation for the cluster Late using DAVID [69] and interaction networks using the Kegg pathways [70]. We applied a Bonferroni correction to account for multiple tests performed. The functional annotation functionality associated gene ID with a biological term which belongs to one out of the 40 annotation categories available in DAVID. DAVID functional annotation clustering measured relationships among the annotation terms based on the share of common genes.
(0.05 MB DOC)
