# Supplementary material for: Gene Expression Profiles Characterize Inflammation Stages in the Acute Lung Injury in Mice
Source: PLoS One. 2010 Jul 8;5(7):e11485. doi: 10.1371/journal.pone.0011485 (PMC2900209; doi:10.1371/journal.pone.0011485)
Supplement: Data file S1 — Comparison of FDR5% and FDR0% to identify significantly differentially expressed genes. In our study, we applied a false discovery rate of 5%, which yielded to a set of 1000 genes significantly differentially expressed across the time­course of our experiment. By filtering our data based on q<0.01 and FDR 5%, we end up with 121 genes significantly differentially expressed in our experiment. By applying a false discovery rate of 0%, we identify 121 genes significantly differentially expressed across the time­course and identical to the ones identified with FDR 5% and q<0.01. (0.14 MB PDF) [file pone.0011485.s001.pdf]

In our study, we applied a false discovery rate of 5%, which yielded to a set of 1000 genes significantly differentially expressed across the time-course of our experiment.
By selecting among these 1000 genes, the ones with $q<0.01$ and FDR 5%, we ended up with 121 genes significantly differentially expressed in our experiment (see supplementary data file #4).
At the same time, we applied a false discovery rate of 0% and then identified 121 genes significantly differentially expressed across the time-course and identical to the ones identified with FDR 5% and $q<0.01$.
We performed unsupervised hierarchical clustering on these 121 genes and identified 3 clusters:

–cluster 1: genes over-expressed at 1-1H30

–cluster 2: genes transitionally over-expressed at 3-4H

–cluster 3: genes over-expressed at 18-24H

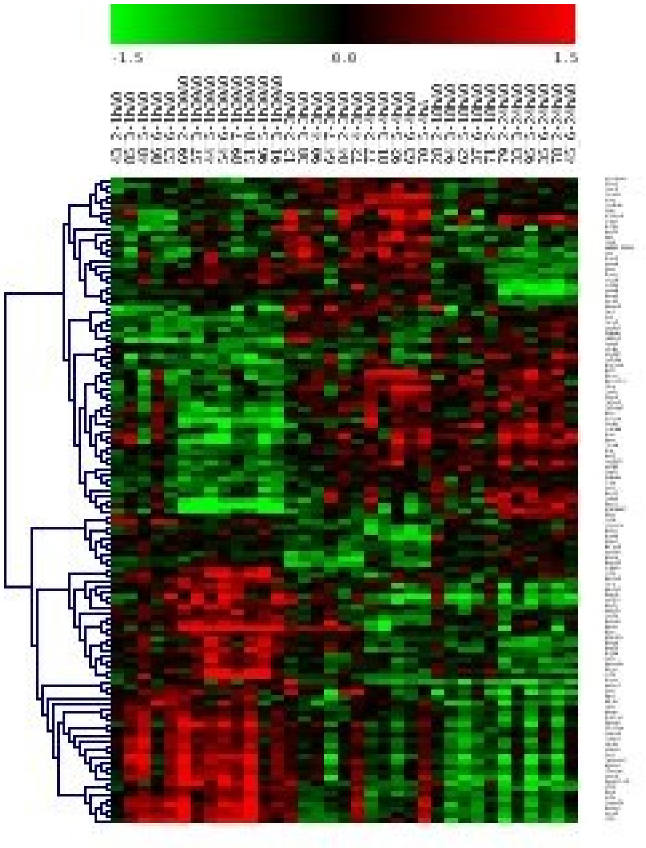

To analyze functional annotation of genes belonging to each cluster, we used DAVID V6.7. In cluster Early, GO terms related immune response such as to regulation of T cell differentiation (3,2.10-3) and lymphocyte activation (1,3.10-2) were highly represented.
Most of the genes grouped in the cluster Intermediate were found to be involved in regulation of transcription (7,9.10-3).
Finally, functional annotation analysis of genes belonging to the cluster Late showed a slight over-representation of GO terms related to chromatin modification (7,4.10-2).

We can conclude that, by limiting our analysis to a very small set of genes (121 genes with FDR5% and $q<0.01$ = 121 genes with FDR 0%), we lost most of the information initially contained in the 1000 genes dataset filtered with FDR 5%. It has to be noted that, using FDR 5% and $q<0.01$, the 121 genes are still grouped into 3 clusters. Functional annotations of the 121 genes are very unspecific and could be found in many other studies. Furthermore, several pathways such as modification of lipid metabolism and migration of the inflammatory signal, which are meaningful because supported by previous studies, are swallowed up by very general descriptions.
