# Supplementary material for: Gene Expression Profiles Characterize Inflammation Stages in the Acute Lung Injury in Mice
Source: PLoS One. 2010 Jul 8;5(7):e11485. doi: 10.1371/journal.pone.0011485 (PMC2900209; doi:10.1371/journal.pone.0011485)
Supplement: Data file S3 — Identification of clusters of samples using hierarchical clustering. Prior to partition samples ( = microarrays) into 3 classes (early, intermediate and late), we performed a hierarchical clustering of the samples in TMEV V4.3.01. using average linkage clustering metrics and Pearson correlation for the distance. This analysis partitioned the samples into 3 groups: early (1H­1H30), intermediate (3H­4H) and late (18H­24H). (0.14 MB PDF) [file pone.0011485.s003.pdf]

Prior to partition samples (=microarrays) into 3 classes (early, intermediate and late), we performed a hierarchical clustering of the samples in TMEV V4.3.01. using average linkage clustering metrics and Pearson correlation for the distance.

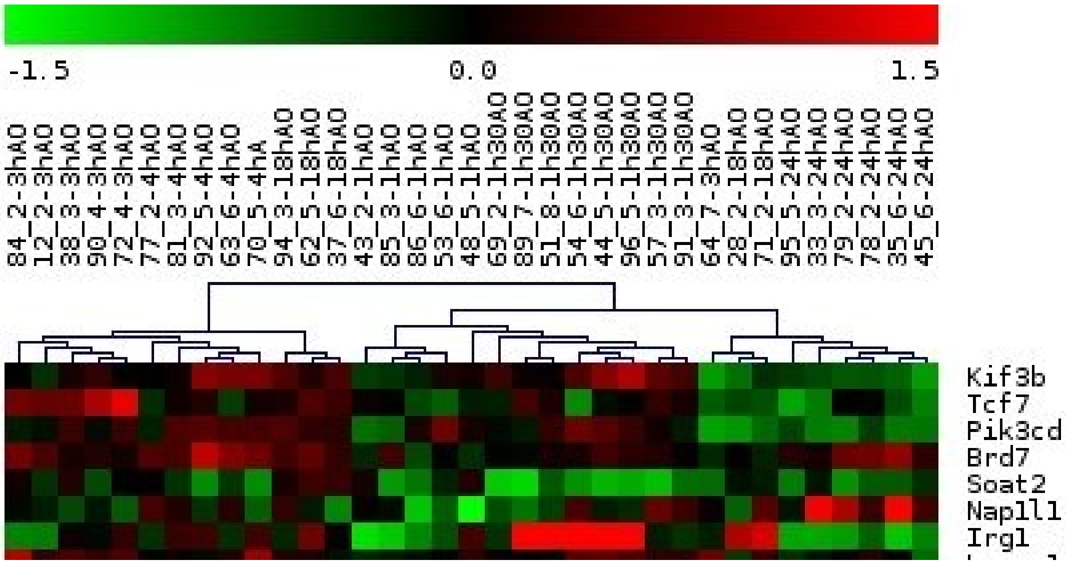

We can see that samples at 1H and 1H30 clustered together. The same way, samples at 3H and 4H clearly clustered together. Finally, samples at 18H and 24H clustered together with the exception of three 18H samples which have been grouped with the 3-4H cluster as an out-group.

This result support our decision to partition the samples into 3 groups: early (1H-1H30), intermediate (3H-4H) and late (18H-24H).
