# Supplementary material for: Gene Expression Profiles Characterize Inflammation Stages in the Acute Lung Injury in Mice
Source: PLoS One. 2010 Jul 8;5(7):e11485. doi: 10.1371/journal.pone.0011485 (PMC2900209; doi:10.1371/journal.pone.0011485)
Supplement: Table S1 — Functional annotation and enrichment of the 366 genes belonging to cluster Late. We identified biological annotation for the cluster Late using DAVID [69] and interaction networks using the Kegg pathways [70]. We applied a Bonferroni correction to account for multiple tests performed. The functional annotation functionality associated gene ID with a biological term which belongs to one out of the 40 annotation categories available in DAVID. DAVID functional annotation clustering measured relationships among the annotation terms based on the share of common genes. (0.05 MB DOC) [file pone.0011485.s005.doc]

**Table S1**. Functional annotation and enrichment of the 366 genes belonging to cluster Late.

| **Functional classification** | |  |  |
| --- | --- | --- | --- |
| *Database* | *Term* | *Count* | *P-Value* |
| Gene ontology | DNA metabolic process | 32 | 7.10E-04 |
| Gene ontology | Regulation of transcription | 63 | 1.10E-03 |
| Gene ontology | Transcription. DNA-dependent | 60 | 1.30E-03 |
| Gene ontology | Regulation of transcription. DNA dependent | 59 | 1.40E-03 |
| Gene ontology | Transcription | 65 | 1.40E-03 |
| Gene ontology | Metabolic process | 186 | 2.20E-03 |
| Gene ontology | Cholesterol metabolic process | 7 | 9.30E-03 |
| **Annotation Cluster 1** | **Enrichment score: 2.07** |  |  |
| *Database* | *Term* | *Count* | *P-Value* |
| Gene ontology | Regulation of nucleobase, nucleoside, nucleotide and nucleic acid metabolic process | 65 | 7.70E-04 |
| Gene ontology | Regulation of transcription | 63 | 1.10E-03 |
| Gene ontology | Transcription, DNA-dependent | 60 | 1.30E-03 |
| Gene ontology | Regulation OF transcription, DNA-dependent | 59 | 1.40E-03 |
| Gene ontology | Transcription | 65 | 1.40E-03 |
| Gene ontology | Regulation of gene expression | 65 | 3.20E-03 |
| Gene ontology | Transcription factor activity | 26 | 4.30E-02 |
| Gene ontology | Transcription regulator activity | 34 | 9.00E-02 |
| **Annotation Cluster 2** | **Enrichment score: 1.96** |  |  |
| *Database* | *Term* | *Count* | *P-Value* |
| Gene ontology | Nucleobase, nucleoside, nucleotide and nucleic acid metabolic process | 93 | 1.90E-03 |
| Gene ontology | Metabolic process | 186 | 2.20E-03 |
| Gene ontology | Cellular metabolic process | 173 | 2.30E-03 |
| Gene ontology | Primary metabolic process | 172 | 4.50E-03 |
| Gene ontology | Macromolecule metabolic process | 145 | 5.00E-02 |
| Gene ontology | Cellular process | 229 | 7.10E-02 |
| **Annotation Cluster 3** | **Enrichment** s**core: 1.44** |  |  |
| *Database* | *Term* | *Count* | *P-Value* |
| Gene ontology | Cholesterol metabolic process | 7 | 9.30E-03 |
| Gene ontology | Sterol metabolic process | 7 | 1.10E-02 |
| Gene ontology | Steroid metabolic process | 9 | 1.80E-02 |
| Gene ontology | Cellular lipid metabolic process | 20 | 5.20E-02 |
| Gene ontology | Alcohol metabolic process | 12 | 6.70E-02 |
| Gene ontology | Lipid metabolic process | 21 | 9.40E-02 |
